# Immediate clinical and neuromechanical effects of cervical spine manipulation compared to cervical spine mobilization among individuals with chronic neck pain: a randomized mechanistic cross-over trial

**DOI:** 10.1186/s12998-026-00674-8

**Published:** 2026-09-07

**Authors:** Salma Bouqartacha, Danja Conconi, Yves Schwendenmann, Mathieu Piché, Petra Schweinhardt, Martin Descarreaux, Lindsay M Gorrell

**Affiliations:** 1 Department of Human Kinetics, Université du Québec à Trois-Rivières, Trois-Rivières, Canada; 2 Department of Chiropractic Medicine, Integrative Spinal Research Group, Balgrist University Hospital, University of Zurich, Zurich, Switzerland; 3 Department of Anatomy, Université du Québec à Trois-Rivières, Trois-Rivières, Canada

**Keywords:** Spinal manipulation, Spinal mobilization, Chronic neck pain, Clinical effects, Pain, Range of motion, Electromyography, Pressure pain threshold, Grip-strength

## Abstract

**Background:**

Spinal manipulation (SM) and spinal mobilization (Smob) are conservative therapies recommended by several clinical guidelines for the treatment of chronic neck pain (CNP). The purpose of this study was to investigate differences between clinical and neuromechanical effects of SM and Smob in patients with CNP.

**Methods:**

This study was a randomized mechanistic cross-over trial. Eighty-nine individuals with mechanical neck pain participated in two sessions, 72 h apart, where they randomly received either SM or Smob during the first session and the other intervention during the second session. Recruitment took place in Zurich from August 2023 to January 2024, and in Trois-Rivières from March 2024 to June 2024. Cervical range of motion (ROM), pain intensity, grip-strength and pressure pain threshold (PPT) outcomes were assessed before and after each treatment, while muscular activity was recorded during the treatment using surface electromyographic electrodes (EMG). PPT and EMG activity were assessed for three muscles bilaterally: sternocleidomastoid (SCM), upper trapezius (UT) and tibialis anterior. Outcomes following SM and Smob were compared using linear mixed-effects model, Wilcoxon signed-rank test and McNemar test.

**Results:**

All ROM increased significantly over time after SM and Smob with no difference between interventions. However, pain intensity analyses showed a significant time x intervention interaction indicating a greater improvement following SM (mean decrease: 0.786 NPRS points) compared with Smob (mean decrease: 0.373 NPRS points) (*β* = 0.413, 95% CI [0.160; 0.665], *p* = 0.002). Muscular response was significantly higher after SM compared to Smob for both SCM (0.144 ± 0.121 vs. 0.023 ± 0.013) and UT (0.083 ± 0.100 vs. 0.024 ± 0.025) muscles, on both sides. A higher PPT was observed for SCM and UT after both interventions. No consistent change was observed for grip-strength. Overall, results showing statistical significance did not reach clinical significance.

**Conclusion:**

Although SM and Smob both led to higher ROM, SM resulted in immediate lower subjective pain intensity and yielded higher muscular responses for participants with CNP. However, given the mechanistic nature of this study and the absence of clinically significant findings, caution is warranted when considering implications for clinical practice.

**Trial registration:**

This trial was registered with Open Science Framework (OCF) on May 23, 2023. https://doi.org/https://doi.org/10.17605/OSF.IO/UNBJ5.

## Background

Neck pain is a common musculoskeletal condition and a major contributor globally to pain, disability, and economic burden [1]. Chronic neck pain (CNP) prevalence has increased by 77.3% between 1990 and 2020 and is expected to rise by 32.5% by 2050 [1], mainly due to population aging [2]. Neck pain has been one of the leading causes of years lived with disability (YLDs) since 1990 [1]. According to the 2019 Global Burden of Disease study, neck pain ranked 11th out of 369 conditions for YLDs [1]. The YLDs associated with CNP increased by 76.2% between 1990 and 2020 [1].

Spinal manipulation (SM) and spinal mobilization (Smob) are two conservative manual therapy techniques recommended by several clinical practice guidelines as part of multimodal approaches for the treatment of CNP [3–6]. While SM is defined as a procedure using high-velocity low-amplitude (HVLA) force characterized by a thrust delivered to an articulation [7], Smob is characterized by a slower oscillatory force with a variable amplitude applied to an articulation [8]. Both SM and Smob have been shown to improve clinical outcomes, with reductions in pain intensity and increases in cervical spine ranges of motion (ROM) observed at short- and intermediate-term follow-up [9–14]. A Cochrane review showed that several studies report similar clinical effects of SM and Smob when used to treat patients with CNP [11].

Evidence comparing the neuromechanical effects of cervical SM and Smob in patients with chronic neck pain is still scarce [12]. It is suggested that both SM and Smob result in increased muscular activation, as measured by electromyography (EMG), although the amplitude and timing of these responses differ between techniques [12, 15–21]. When delivered to the thoracic spine, muscular response during and after Smob was shown to be two to three times lower compared to SM [17, 20]. This difference has been suggested to be partly explained by differences in force–time characteristics of the two interventions [17, 19, 20, 22]. In addition to muscular activity, studies investigating the effect of SM and Smob on pressure pain thresholds (PPT) have suggested that both interventions can lead to increases in PPT, immediately and locally, at the site of the intervention [13, 20, 23, 24]. Two systematic reviews reported an increased PPT following either cervical SM [23] or cervical Smob [23, 24]. Grip-strength has been identified as an indicator of both muscular function and overall physical condition [25] and low grip-strength score is correlated with several diseases (e.g., cardiovascular disease, sarcopenia, fragility fractures). However, only a few studies have explored the relationship between grip-strength and cervical SM or cervical Smob, and while the results are conflicting, no significant change in grip-strength following SM has been reported among adults with CNP [26, 27].

The similarities in clinical and neuromechanical effects of SM and Smob may partly be driven by the use of a general term in clinical research to describe both interventions: spinal manipulative therapy [20]. This lack of precision has led to broad interpretations and insufficient quality evidence for clinicians to make treatment decisions about the specific use of SM or Smob [20]. Direct comparison between cervical SM and cervical Smob would be helpful to inform patient-centered intervention decisions. Therefore, the objective of this mechanistic study was to explore the differences between immediate clinical and neuromechanical effects of cervical SM and cervical Smob among patients with CNP.

We hypothesized that cervical SM and cervical Smob would lead to different immediate results in all clinical and neuromechanical outcomes. However, the extent of those differences in terms of statistical and clinical relevance remain to be determined.

## Methods

### Design

This study was a randomized mechanistic cross-over trial conducted at two biomechanics research laboratories: one at Balgrist University Hospital (BUH) (Switzerland) and the other at the Université du Québec à Trois-Rivières (UQTR) (Canada). Ethical approval for the project was obtained from the Cantonal Ethics Committee of Zurich (BASEC no. 2023-00997) and from the Research Ethics Board for human research at UQTR (CER-23-302-10.01). All participants provided written informed consent at both sites.

### Participants

Eighty-nine participants with CNP were recruited from the local populations of Zurich (Switzerland) and Trois-Rivières (Canada). The recruitment took place in Zurich from August 2023 to January 2024, and in Trois-Rivières from March 2024 to June 2024. Recruitment strategies included the use of flyers and posters that were distributed among local populations of both sites and displayed in the university and university clinics in Trois-Rivières. Moreover, social media platforms were used to target eligible individuals. An initial eligibility screening was conducted by a research assistant through a phone call with individuals who showed interest by either calling or emailing the research team. Inclusion and exclusion criteria were discussed and assessed with potentially eligible participants, and those who met all inclusion criteria were enrolled in the study. Inclusion and exclusion criteria can be found in Table 1.

**Table 1:** Inclusion and exclusion criteria

Inclusion criteria	Exclusion criteria
Age 18–65 years Ability to understand and communicate in French, English or German Mechanical neck pain located below the superior nuchal line and inferior border of the mandible and above the superior border of the clavicle, suprasternal notch and scapular spines bilaterally, with a duration of ≥ 12 weeks [28]	Neck pain not originating from the cervical spine Conditions or medications that could affect heart rate variability signals (e.g., hypertension, diabetes, cardiovascular disease, obesity (BMI > 30), pregnancy, current use of pain medications, steroids, antidepressants or β-blockers) and contraindications to the application of SM and Smob (e.g., possibility of cerebrovascular compromise, osteoporosis, personal or family history of connective tissue disorders, current use of anticoagulant therapy, history of recent surgery or neck trauma, facial or intra-oral anesthesia or paresthesia, visual disturbances, dizziness or vertigo) Participants who received any manual treatments (e.g., SM, Smob, therapeutic massage) less than two weeks prior to data collection [29, 30] Participants who took any type of pain medication 24 h prior to each data collection session

BMI: Body Mass Index; SM: spinal manipulation; Smob: spinal mobilization

### Experimental protocol

#### Timeline and clinical assessment

**Fig. 1:**
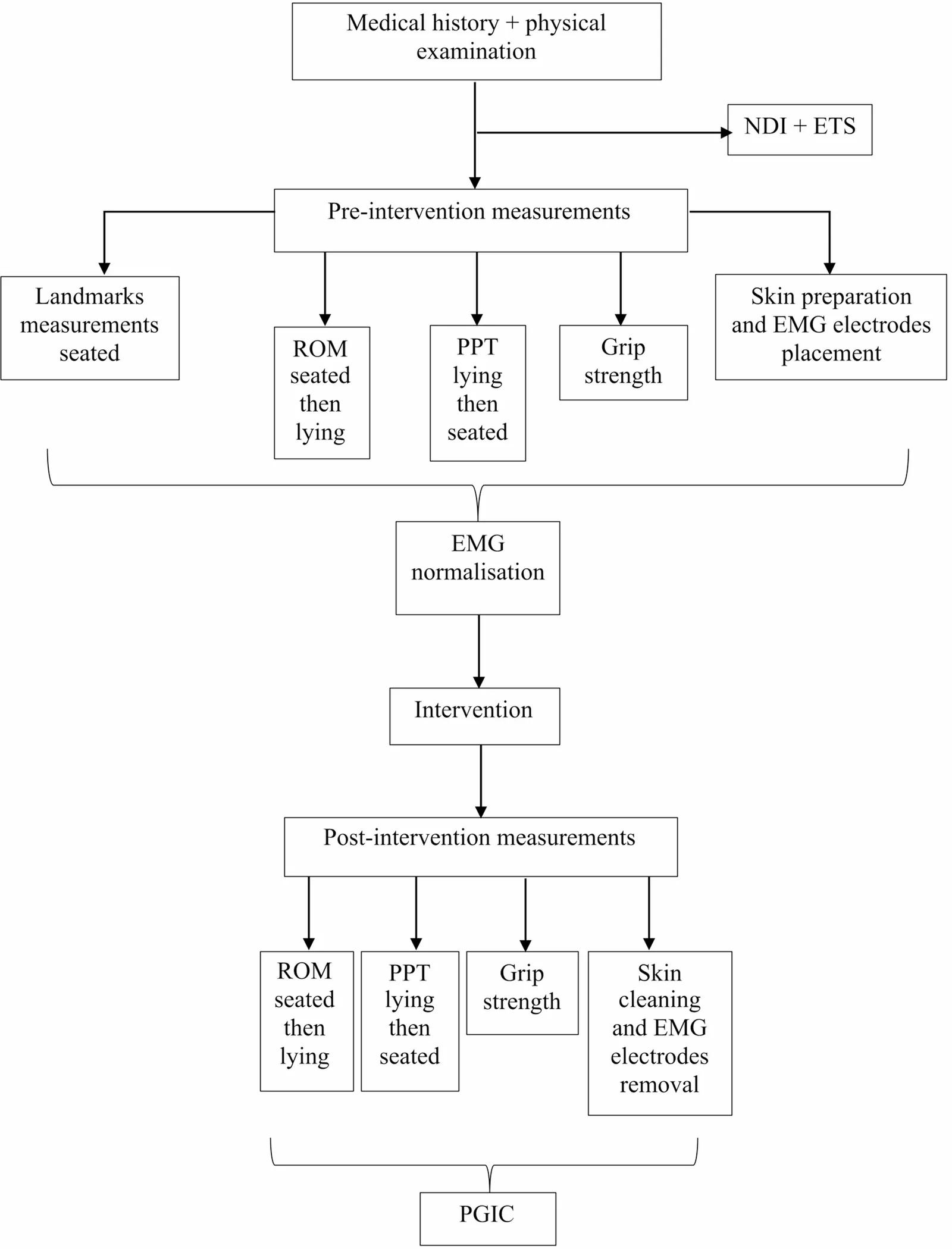
Data collection flow chart for a typical session. ROM: Range Of Motion, PPT: Pressure Pain Threshold, EMG: electromyography, NDI: Neck Disability Index, ETS: Expectation of Treatment Scale, PGIC: Patient Global Impression of change

The experiment followed a within-subject crossover design and consisted of two sessions, conducted 72 h apart, and each lasting two to three hours. Participants were randomly assigned to receive either a cervical SM or a cervical Smob during the first session and the other intervention at the second session. At the beginning of each session, a short targeted medical history and physical examination were carried out by a clinician to rule out any red flags or contraindication to SM or Smob. In Zurich, the clinician was either a registered and practicing chiropractor with 10 years of experience, or a registered chiropractor pursuing graduate studies, whereas in Trois-Rivières, the clinician was a registered chiropractor pursuing graduate studies. During physical examination, the chiropractor located and marked the level and side of the most painful cervical vertebra, which would later serve as a landmark for clinicians delivering the interventions (the same vertebral level was targeted to deliver the intervention on both sessions). Participants were then asked to complete two questionnaires before pre-intervention measurements: i) Neck Disability Index (NDI) [31] and ii) Expectation of Treatment Scale (ETS) [32]. Pre-intervention measurements included ROM, PPT and grip-strength. EMG normalization was then completed and followed by the intervention. Post-intervention measurements then followed in the same order as pre-intervention measurement—ROM, PPT and grip-strength. Finally, participants were asked to complete the Patient Global Impression of Change (PGIC) questionnaire [33] (see Fig. 1 for the detailed flow chart).

#### Interventions

SM was delivered by registered practicing chiropractors at both sites. Smob was delivered by registered chiropractors and physiotherapists at BUH, and by registered manual therapists (chiropractors, osteopath, masso-kinesiotherapist with a manual therapy degree) at UQTR. All clinicians from BUH had more than five years of clinical experience and clinicians from UQTR had two or more years of clinical experience.

SM consisted of a single high-velocity, low-amplitude thrust within the sagittal plane and along a posterior-to-anterior linear vector, delivered to the targeted cervical zygapophyseal joint of the participant in a supine position. Smob consisted of repeated mobilizations (30 s) applied to the joint articulation using a bony contact on the spinous process of the targeted cervical vertebra in a posterior-anterior motion with the participant in a prone position [34]. The second session was conducted in the exact same way, except for the intervention depending on the randomization order (see Fig. 1 for the detailed flow chart). As this mobilization procedure was never performed by chiropractors at BUH and none of the clinicians at UQTR, they were trained by an experienced (18 years) Orthopaedic Manual Physical Therapy physiotherapist from BUH (on site at BUH and by a videocall meeting at UQTR) who regularly delivered this type of prone mobilizations in their practice. The standardized application of Smob was adhered to by all clinicians, regardless of their clinical training.

During the intervention, to minimize expectation bias and other non-specific effects, clinicians had no interaction with participants other than introducing themselves and delivering the allocated intervention. For cervical SM, the clinician was handed a flexible pressure mat to put between their index finger (as the contact for the thrust) and the participant’s neck. For cervical Smob, the clinician was handed the flexible pressure mat to put between their thumbs (as the contact for the mobilizations) and the participant’s neck. The clinician was then asked to perform repeated mobilizations, for 30 s, to the cervical spine level identified with the landmark.

#### Force–time characteristics of SM and Smob

A pliance-Novel measuring system (Novel, pliance-xf-16 Medical and accessories, Germany) was used, with a customized high-resolution pressure mat (Pliance, Novel, Germany), allowing for the measurement of the forces applied by the clinician at the clinician-participant interface (cervical spine) with a recording frequency of 200Hz. The pressure mat measured 10 cm × 5 cm × 0.02 cm and consisted of a matrix of 50 sensors, each measuring 1 cm × 1 cm.

The recorded forces allowed an initial visual assessment of the time-force profiles of SM and Smob during data collection and enabled determination of the peak force and the peak time of force, which were further used during EMG data analysis (see statistical analysis section). A low-pass filter with a cutoff frequency of 5Hz was applied to the Smob force signal to identify cycle peak forces. The peak force was defined as the highest force value reached during SM thrust or Smob oscillations, whereas the time to peak force was defined as the time between the instant pressure was applied by the clinician at the beginning of the procedure and the peak force [22, 35, 36].

### Adverse events

The nature of any adverse event, occurring during and/or immediately following the session, in addition to the severity (mild, moderate or severe) were documented at each session using a standardized reporting form. The nature of adverse events was first categorized into the following categories: i) unfavorable or unintended finding, ii) unfavorable or unintended symptom or iii) unfavorable or unintended disease. If a serious adverse event were to occur, it was categorized as: i) resulted in death or was life-threatening, ii) required in-patient hospitalization or prolongation of existing hospitalization, iii) resulted in persistent or significant disability or incapacity or iv) caused a congenital anomaly/birth defect. All adverse events were reported to the responsible ethical authority.

### Randomization and blinding

Participants were randomly allocated to the order of intervention using a stratified randomization procedure. Stratification was performed according to age and sex. This approach ensured balanced group allocation across age and sex strata.

Participants were told that the interventions used in the study are recommended and widely used treatments for neck pain. However, blinding of participants was not feasible in the context of this study. Participants could likely distinguish between the interventions due to their different procedural characteristics (HVLA thrust versus repeated oscillatory mobilizations). Clinicians who performed the interventions were blinded to the study hypothesis and were not involved in pre or post-intervention measurements. Two researchers were involved during data collection sessions. One researcher was responsible for intervention allocation and coordination with the clinicians, whereas the second researcher performed outcome assessments and was not informed of intervention allocation until after data collection was completed at the end of the second session. To maintain blinding of the outcomes’ assessor, the second researcher always left the room prior to clinician entry and returned only once participants were repositioned in a seated position following the intervention, thereby preventing knowledge of the intervention delivered. However, the force–time profiles were identifiable during subsequent data processing and analysis.

### Clinical and neuromechanical outcomes

Active ROM, PPT and grip-strength were assessed using a JTech Medical system (JTech Medical and accessories, Midvale, Utah, USA) during pre- and post-intervention measurements. PPT and EMG responses were assessed on three muscles bilaterally: sternocleidomastoid (SCM), upper trapezius (UT) and tibialis anterior (TA). The primary outcome was pain intensity and the secondary outcomes were ROM, PPT, grip-strength and EMG.

#### Pain intensity

Pain intensity was assessed during the medical history and after post-intervention measurements with a numerical pain rating scale (NPRS). Participants were asked to rate their current pain intensity on a scale from 0 = no pain to 10 = worst pain imaginable [31].

#### Ranges of motion

Participants were asked to perform active cervical ROM: flexion, extension, bilateral lateral-flexion and bilateral rotation, each three times, by “going to the maximum of their range of motion until they reach their pain sensation or discomfort”. An inclinometer (JTech Medical Dualer IQ Pro digital inclinometer, Midvale, Utah, USA) was placed on top of the participant's head (for flexion, extension and bilateral lateral-flexion) and on the forehead (for bilateral rotation) at an equal distance from both ears. Flexion, extension and lateral-flexion were assessed with the participants in a seated position on a chair with their back and head straight, while rotation was assessed in a supine position. The average of repetitions was used for data analysis [37].

#### Pressure pain threshold

Pressure pain threshold was measured using an algometer with a round tip with 1cm diameter (JTech Medical Commander Echo Wireless pain testing algometer starter kit, Midvale, Utah, USA). The location of the PPT measurements depended on the muscle and is listed in Table 2. Perpendicular pressure was applied to the muscle with a constant speed of 0.5 kg/s. A metronome set to 60 beats per minute was used to guide the application of the pressure. Participants were asked to say “STOP” once they “first perceived a change in the quality of the pressure such as burning, stinging, drilling or aching” [38]. To familiarize participants with the change in sensation, three repetitions were conducted on the thenar of the dominant hand. PPT were then assessed for each muscle three times, with a ten second rest between tests and alternating between the right and the left side [26, 39]. The three trials were averaged, and the mean was used for data analysis [40].

**Table 2:** Pressure pain threshold measurements

Muscle	PPT
Tibialis anterior	Five centimeters distal to anterior tibial tuberosity (ATT) on the line ATT-electrode placement [41]
Sternocleidomastoid	Proximal insertion right below the mastoid process [41]
Upper trapezius	Mid-point between the spinous process of the 7th cervical vertebra and the acromion [41]

ATT: Anterior tibial Tuberosity

#### Grip strength

Grip strength was measured using a hand-grip dynamometer (JTech Medical Commander Echo grip dynamometer starter kit, Midvale, Utah, USA). Participants were seated on a chair with their elbow and wrist at a 90° angle supported by the arm rest. They were then handed the device and asked to squeeze it to the maximum of their ability, three times for each hand, while alternating sides. The researcher encouraged them to squeeze the hand-grip to their maximum ability. The mean score obtained from the three trials was used for data analysis [42].

#### Muscular activity and normalization

Muscular activity was recorded using wireless bipolar surface EMG electrodes (circular shape, 41×15.5x11.3mm, Pico EMG, Cometa Systems, Italy). Landmark measurements for electrodes placements were defined according to the current SENIAM recommendations for sensor locations on individual muscles for UT and TA, and according to the recommendations by Falla et al*.* for SCM [43]. All electrodes were placed parallel to the muscle fibers, with an interelectrode distance greater than 20mm. To reduce impedance, skin was shaved and cleaned with fine sandpaper (Red DotTrace Prep; 3M, St. Paul, MN) or with a scrubbing paste (NuPrep skin preparation gel, Weaver & Co., Aurora, CO, USA) and then with alcohol (70% ethyl alcohol).

To reduce inter- and intra-individual variability, normalization was performed and the maximum voluntary contraction (MVC) was chosen considering the *Consensus for Experimental Design in Electromyography* project [44]. Participants were asked to actively resist to the maximum of their ability against the researcher’s force for five seconds. The position of the participant depended on the tested muscle. Normalized EMG data were used for all data analyses.

### Questionnaires

Participants were asked to complete two questionnaires before both pre-intervention measurements: Neck Disability Index (NDI) [31] and Expectation of Treatment Scale (ETS) [32]. At the post-intervention measurements, participants completed the Patient’s Global Impression of Change (PGIC) questionnaire [33]. These questionnaires were included to assess participants’ baseline disability and treatment expectations, as well as to capture participants’ global impression of change following the interventions.

### Sample size

For this cross-over, the sample size calculation was based on a paired t-test within-subject comparison of pain (participants received both interventions). Assuming a two-sided hypothesis, an alpha level of 0.05, a beta level of 0.20, and a mean difference in pain of 1.3 [31], 41 participants were required. To account for an anticipated attrition and missing data rate of 15%, the target sample size was increased to a minimum of 50 participants.

Pragmatic considerations were taken as data collection was conducted across two sites internationally and within a broader research program that also included additional planned analyses and control participants. Recruitment targets were therefore considered to ensure that the minimum targeted sample size would be achieved within each data collection site.

### Data analysis

Customized MATLAB (R2023b Update 6 (23.2.0.2485118)) scripts were created for each intervention (SM and Smob). EMG data were filtered using a band-pass filter (30-450Hz). SM and Smob force–time profiles were used to identify peak forces and peak-times which were subsequently used to calculate EMG activity in response to either SM or Smob. For SM, a time window was generated 250ms before and after the peak-time of the thrust, and the normalized Root Mean Square (nRMS) was then calculated for the three muscles bilaterally. For Smob, a time window was generated 250ms before and after each mobilization cycle’s peak force. The nRMS was then calculated for the three muscles bilaterally and for each cycle, and the average of all nRMS cycle values for each muscle and side was calculated.

### Statistical analysis

The level of statistical significance was set at *p* ≤ 0.05 for all analyses. IBM SPSS Statistics, version 29.0.2.0 [20] and Statistica version 13.5.0.17 (Tibco Statistica 13.5) softwares were used for statistical analysis. Data were checked for normality before any statistical analysis.

Data were analyzed using a linear mixed-effects model to account for the crossover repeated-measures design. The model included fixed effects for treatment (SM vs Smob), time (pre-intervention vs post-intervention), period (first vs second session), and sequence (SM first vs Smob first), as well as the treatment × time interaction. Participants were included as random intercepts to account for within-subject correlation across repeated measurements.

Estimated marginal means were used to describe group means and changes. Degrees of freedom were estimated using the Satterthwaite approximation. Statistical significance was set at *p* < 0.05.

To facilitate results’ interpretations, ROM data were categorized into three 180° arcs of motion: flexion–extension, right and left lateral-flexion, right and left rotation. Wilcoxon signed-rank and McNemar tests were used for the questionnaires: NDI, ETS and PGIC. The Wilcoxon signed-rank test was used to compare muscle responses between SM and Smob. Between-sequence comparisons of nRMS differences were performed using Mann–Whitney test and between-period comparisons of nRMS values using Wilcoxon signed-rank test.

### Handling of missing data and drop-outs

**Fig. 2:**
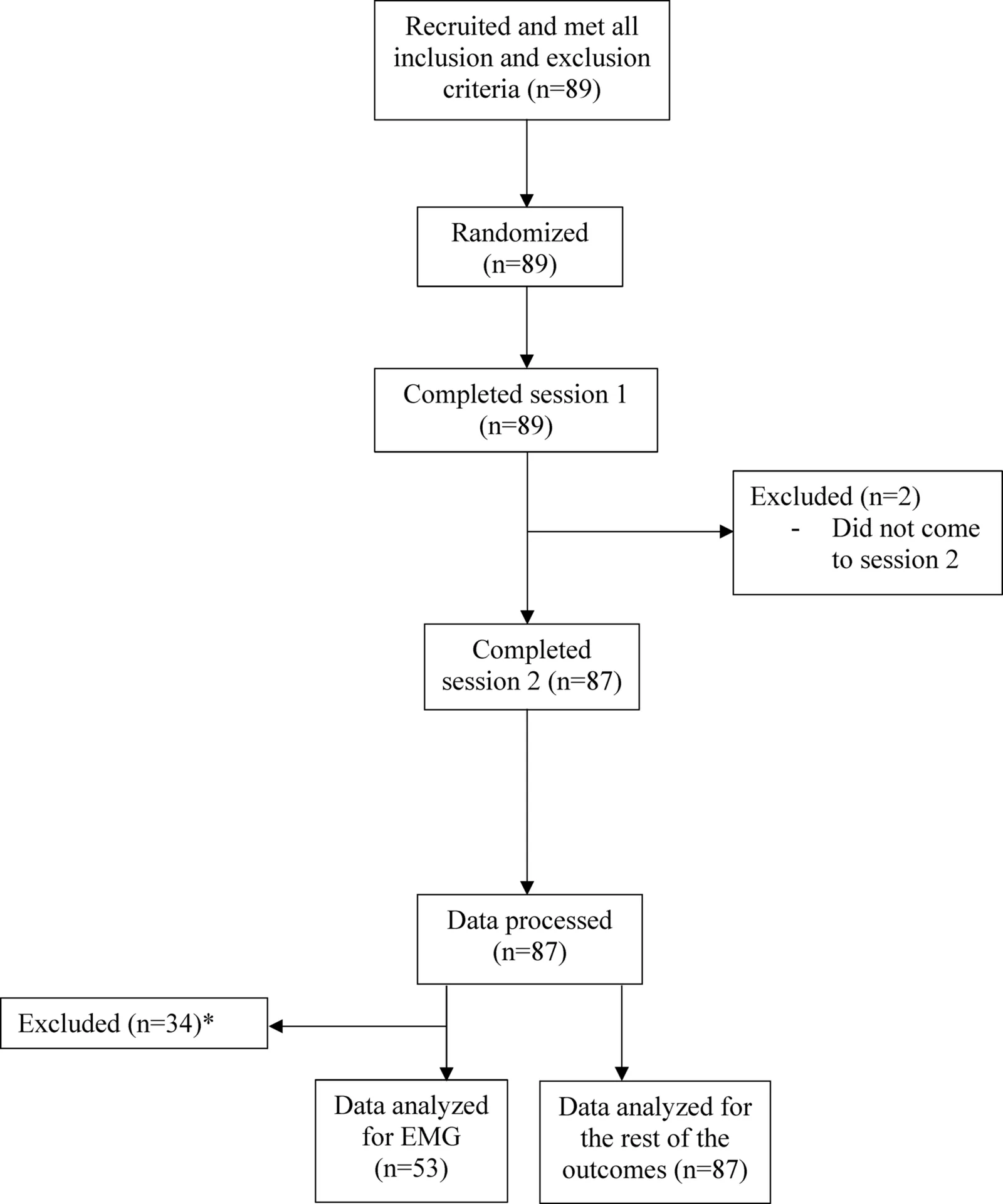
Participants’ flow chart. EMG: electromyography, SM: spinal manipulation, Smob: spinal mobilization. *Due to technical issues affecting the SM and Smob time-event markers

Eighty-nine participants were recruited and randomized. Two participants did not attend the second session and were therefore excluded from all analyses. Overall, data from 87 participants were included in the data analysis except for EMG, for which data were only usable for 53 participants due to technical issues affecting the SM and Smob time-event markers resulting in unusable force–time profiles. Therefore, accurate synchronization of the interventions time-event markers with the EMG signals was not possible, precluding valid analysis for the rest of EMG data (see Fig. 2).

## Results

### Baseline characteristics

The mean age of the sample was 35 years (SD=12) and 65% of participants were female. Based on the NDI questionnaire, 1.1% of participants reported no disability, 78.7% presented mild to moderate disability and 20.2% reported moderate to severe disability. Socio-demographic characteristics, NDI and expectation scores at baseline can be found in Table 3.

**Table 3:** Socio-demographic characteristics, ETS and NDI scores at baseline

Variable	Value
Age (years): mean (SD)	35 (12)
Height (cm): mean (SD)	170 (8)
Weight (kg): mean (SD)	72 (13)
Sex	65% F
NDI Score /50	< 5 (1.1%) → no disability 5–24 (78.7%) → mild to moderate disability ≥ 25 (20.2%) → severe to complete disability

ETS questions:	Partially disagree (%)	Partially agree (%)	Agree (%)	Definitely agree (%)
1. I expect that the treatment will help me deal with my symptoms better	SM: 10.1 Smob: 6.9	SM: 28.1 Smob: 34.5	SM: 37.1 Smob: 35.6	SM: 24.7 Smob: 23.0
2. I expect that the treatment will make my symptoms go away	SM: 32.6 Smob: 35.6	SM: 41.6 Smob: 34.5	SM: 17.9 Smob: 20.7	SM: 7.9 Smob: 9.2
3. I expect my energy to improve with the treatment	SM: 24.7 Smob: 23.0	SM: 32.6 Smob: 35.6	SM: 29.2 Smob: 27.6	SM: 13.5 Smob: 13.8
4. I expect improved physical performance for the treatment	SM: 28.1 Smob: 28.7	SM: 36.0 Smob: 35.6	SM: 23.6 Smob: 21.9	SM: 12.3 Smob: 13.8
5. I expect that my symptoms will improve significantly after the treatment	SM: 22.5 Smob: 24.1	SM: 36.0 Smob: 26.5	SM: 28.0 Smob: 31.0	SM: 13.5 Smob: 18.4

NDI: Neck Disability Index, SD: Standard Deviation, SM: spinal manipulation, Smob: spinal mobilization, ETS: Expectation of Treatment Scale, kg: kilograms, cm: centimeters, F: female

### Adverse events

No serious adverse events were reported following the interventions. One participant experienced dizziness during physical examination, prior to any intervention, was evaluated by paramedics and referred for further assessment.

### Questionnaires

There was no difference in NDI at the beginning of each session (*Z* = 0.61, *p* = 0.545). Although the median score of PGIC was 3 for both sessions, a significant difference (*Z*= 3.31, *p* <0.001) in the distribution of data was observed in favor of SM, showing an interquartile range (*Q1-Q3*) of 2–3 for SM and 3–4 for Smob. This difference did not reach the minimal clinically important difference (MCID) threshold (score ≤ 2) [45]. Expectations, measured by the ETS, showed no difference between both interventions (*p* >0.050).

### Pain intensity

**Fig. 3:**
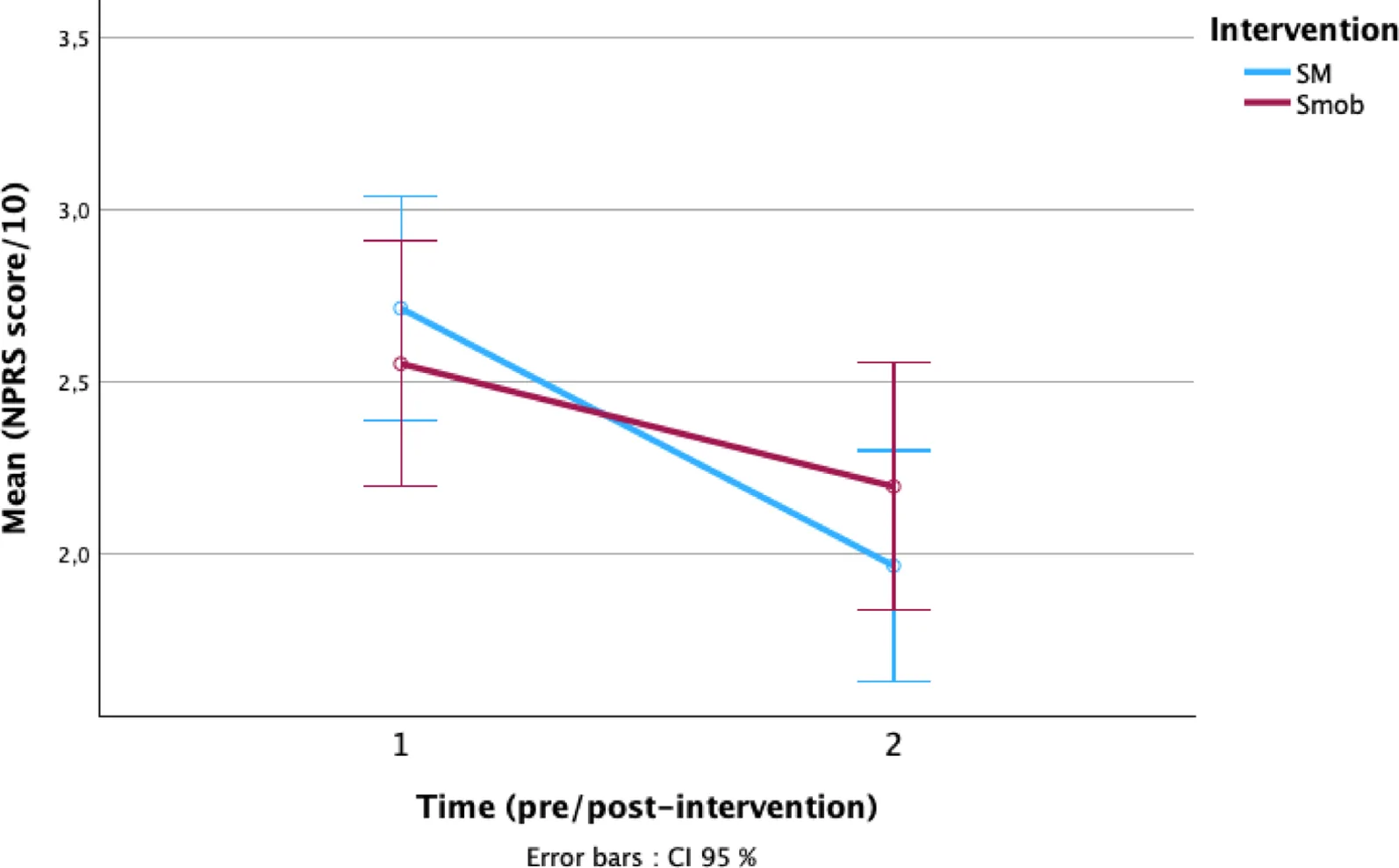
Pain intensity pre- and post-intervention (SM vs Smob). SM: Spinal Manipulation, Smob: Spinal mobilization, CI: confidence interval.

Pain intensity was not significantly different between SM and Smob (main effect: *F* = 0.09, *p* = 0.763). However, pain intensity decreased significantly over time (main effect: *F* = 42.18, *p* < 0.001) and this effect was significantly different between SM and Smob (interaction: *F* = 10.61, *p* = 0.002), indicating a greater improvement following SM (mean decrease: 0.786 NPRS points) compared with Smob (mean decrease: 0.373 NPRS points) (*β* = 0.413, 95% CI [0.160; 0.665], *p* = 0.002). However, this difference did not reach the MCID of 1.3 points on the NPRS [31] (see Fig. 3). Sequence effect was not significant (*F* = 0.008, *p* = 0.928) but a significant period effect (*F* = 5.04, *p* = 0.027) was observed.

### Ranges of motion

Flexion–extension range of motion increased significantly over time (main effect: *F* = 19.55, *p* < 0.001), with improvements of 4.6° after SM and 3.7° after Smob, both reaching the MCID [26]. This range of motion was not significantly different between SM and Smob (main effect: *F* = 0.18, *p* = 0.677) and no significant treatment x time interaction was found (*F* = 0.36, *p* = 0.549). No significant sequence effect (*F* = 0.17, *p* = 0.683) or period effect (*F* = 0.72, *p* = 0.398) was observed.

Similarly, lateral-flexion range of motion increased over time (main effect: *F* = 28.27, *p* < 0.001), with 4° after SM, which reached the MCID [26], and 2.6° after Smob. No significant difference was found between SM and Smob (main effect: *F* = 2.41, *p* = 0.124), and no significant interaction effect was observed (*F* = 1.61, *p* = 0.208). No significant sequence effect (*F* = 0.40, *p* = 0.527) or period effect (*F* = 1.64, *p* = 0.204) was observed.

Finally, rotation range of motion increased as well over time (main effect: *F* = 5.11, *p* = 0.026) increasing 1.6° after SM and 1.4° after Smob*.* No significant difference between SM and Smob was observed (main effect: *F* = 1.93, *p* = 0.168), nor was there a significant interaction effect (*F* = 0.04, *p* = 0.850). Sequence effect was not significant (*F* = 0.16, *p* = 0.690) but a significant period effect (*F* = 8.32, *p* = 0.005) was observed (See Table 4 for detailed results).

**Table 4:** Mean values of the three arcs of range of motion pre- and post-intervention for SM and Smob

	SM	Smob		
	Pre-intervention	Post-intervention	Pre-intervention	Post-intervention
Arc flexion–extension Mean° (*SE*)	115.2 (2.4)	119.8 (2.2)	115.2 (2.4)	118.9 (2.3)
Arc lateral-flexion Mean° (*SE*)	76.0 (2.3)	80.0 (2.4)	77.8 (2.3)	80.5 (2.4)
Arc rotation Mean° (*SE*)	147.5 (2.5)	149.1 (2.5)	146.2 (2.5)	147.6 (2.5)

SM: spinal manipulation, Smob: spinal mobilization, SE: Standard Error, °: degree of ranges of motion

### Pressure pain threshold

Statistical analysis resulted in inconsistent changes depending on the muscle and the side:

#### SCM muscle

**Fig. 4:**
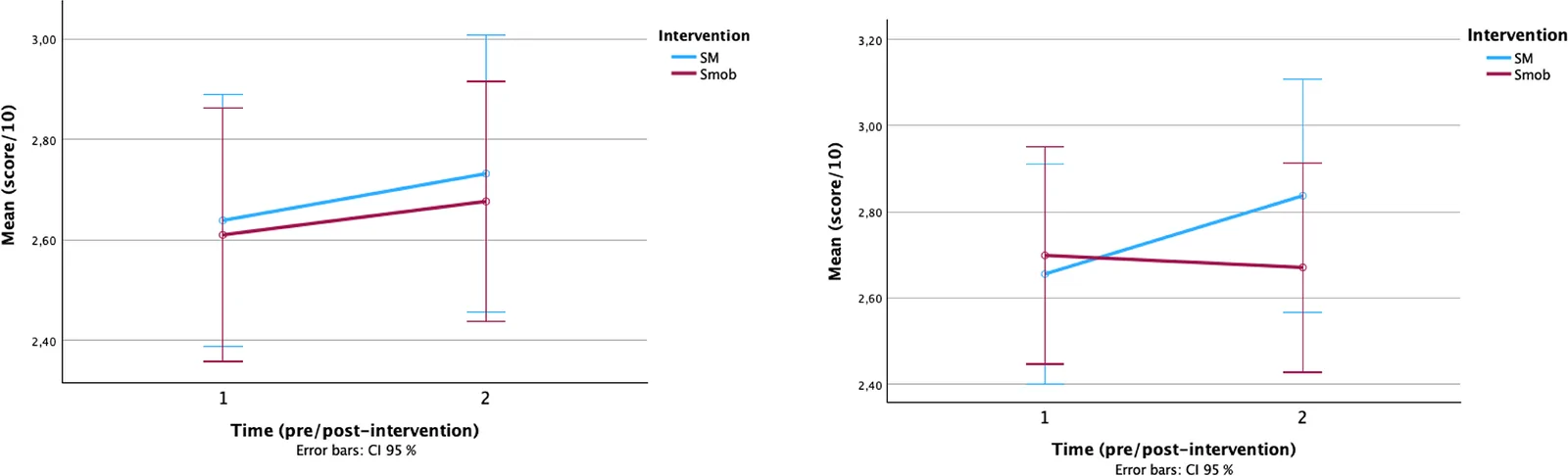
SCM (left side left, right side right) pressure pain threshold pre- and post-intervention (SM vs Smob). SM: Spinal Manipulation, Smob: Spinal mobilization, CI: confidence interval, SCM: sternocleidomastoid.

PPT of the right SCM muscle was not significantly different between SM and Smob (main effect: *F* = 1.02, *p* = 0.316), nor did it change significantly over time (main effect: *F* = 2.47, *p* = 0.120). However, a significant interaction effect was observed (*F* = 8.06, *p* = 0.006) indicating an increase in PPT after SM (mean increase: 0.176 kg) and a decrease in PPT after Smob (mean decrease: 0.029 kg) (*β* = − 0.205, 95% CI [− 0.349; − 0.062], *p* = 0.006). This difference did not reach the smallest detectable difference (SDD) for PPT measured in cervical spine muscles [45]. Sequence effect was not significant (*F* = 0.07, *p* = 0.786) but a significant period effect (*F* = 20.32, *p* < 0.001) was observed (See Fig. 4).

For the left SCM muscle, PPT did not significantly change over time (main effect: *F* = 3.38, *p* = 0.069), nor did it differ between SM and Smob (main effect: *F* = 0.26, *p* = 0.610), and no interaction effect was observed (*F* = 0.09, *p* = 0.763). Sequence effect was not significant (*F* = 0.001, *p* = 0.970) but a significant period effect (*F* = 5.89, *p* = 0.017) was observed (See Fig. 4).

#### UT muscle

**Fig. 5:**
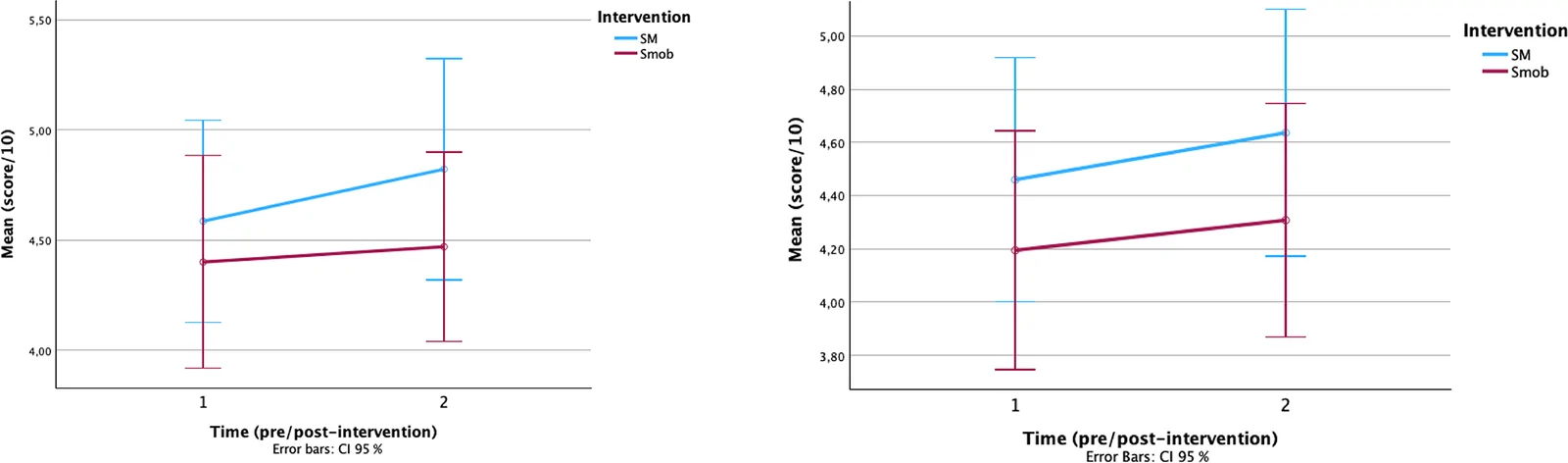
UT (left side left, right side right) pressure pain threshold pre- and post-intervention (SM vs Smob). SM: Spinal Manipulation, Smob: Spinal mobilization, CI: confidence interval, UT: upper trapezius.

PPT of the right UT muscle was significantly different between SM and Smob (main effect: *F* = 5.43, *p* = 0.022). In addition, PPT increased significantly over time (main effect: *F* = 4.98, *p* = 0.028), with increases observed after both SM (mean increase: 0.204 kg) and Smob (mean increase: 0.103 kg). This effect was not significantly different between SM and Smob (interaction: *F* = 0.55, *p* = 0.462). Sequence effect was not significant (*F* = 0.31, *p* = 0.581) but a significant period effect (*F* = 6.45, *p* = 0.013) was observed (See Fig. 5).

For the left UT muscle, PPT did not significantly change over time (main effect: *F* = 3.63, *p* = 0.060), nor did it differ between SM and Smob (main effect: *F* = 2.45, *p* = 0.122), and no interaction effect was observed (*F* = 1.62, *p* = 0.206). No significant sequence effect (*F* < 0.001, *p* = 0.986) or period effect (*F* = 3.81, *p* = 0.054) was observed (See Fig. 5).

#### TA muscle

**Fig. 6:**
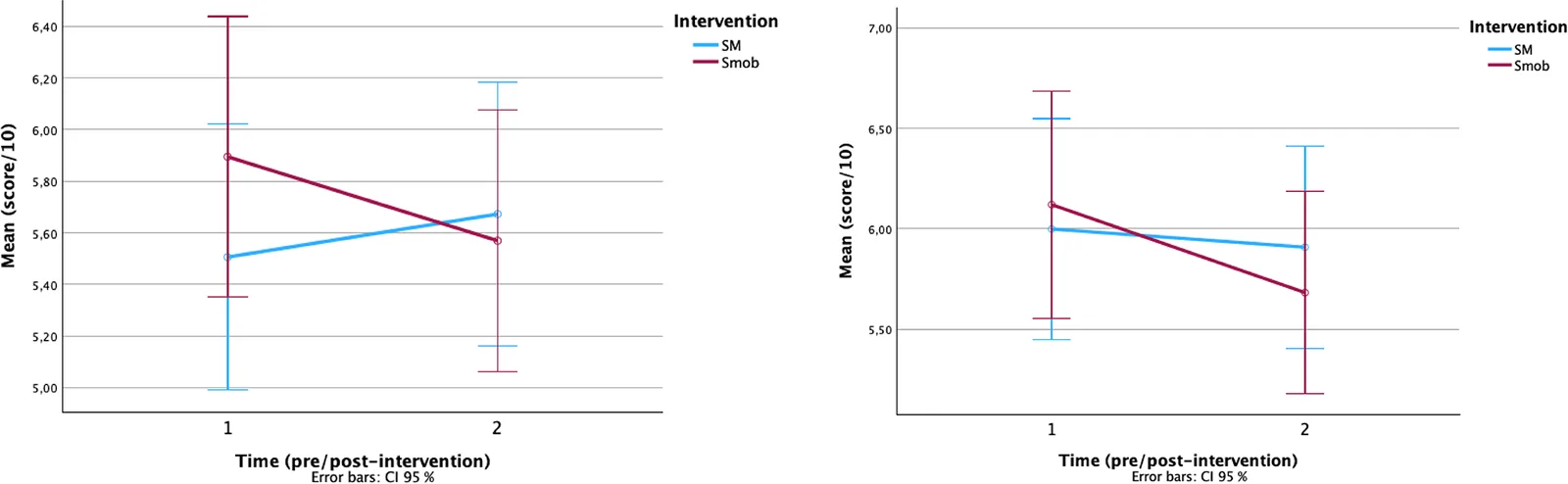
TA (left side left, right side right) pressure pain threshold pre- and post-intervention (SM vs Smob). SM: Spinal Manipulation, Smob: Spinal mobilization, CI: confidence interval, TA: tibialis anterior.

PPT of the right TA muscle decreased significantly over time (main effect: *F* = 5.33, *p* = 0.023), with decreases observed after both SM (mean decrease: 0.055 kg) and Smob (mean decrease: 0.412 kg). PPT was not significantly different between SM and Smob (main effect: *F* = 0.081, *p* = 0.776). Moreover, no interaction effect was observed (*F* = 3.55, *p* = 0.063). No significant sequence effect (*F* = 0.05, *p* = 0.822) or period effect (*F* = 0.26, *p* = 0.609) was observed (See Fig. 6).

For the left TA muscle, PPT did not significantly change over time (main effect: *F* = 0.30, *p* = 0.584), nor did it differ between SM and Smob (main effect: *F* = 1.01, *p* = 0.318). A significant interaction effect was observed (*F* = 6.96, *p* = 0.010) with an increase in PPT after SM (mean increase: 0.215 kg) and a decrease in PPT after Smob (mean decrease: 0.306 kg) (*β* = − 0.520, 95% CI [− 0.912; − 0.128], *p* = 0.010). No significant sequence effect (*F* = 0.13, *p* = 0.715) or period effect (*F* = 0.35, *p* = 0.556) was observed (See Fig. 6).

### Grip strength

**Fig. 7:**
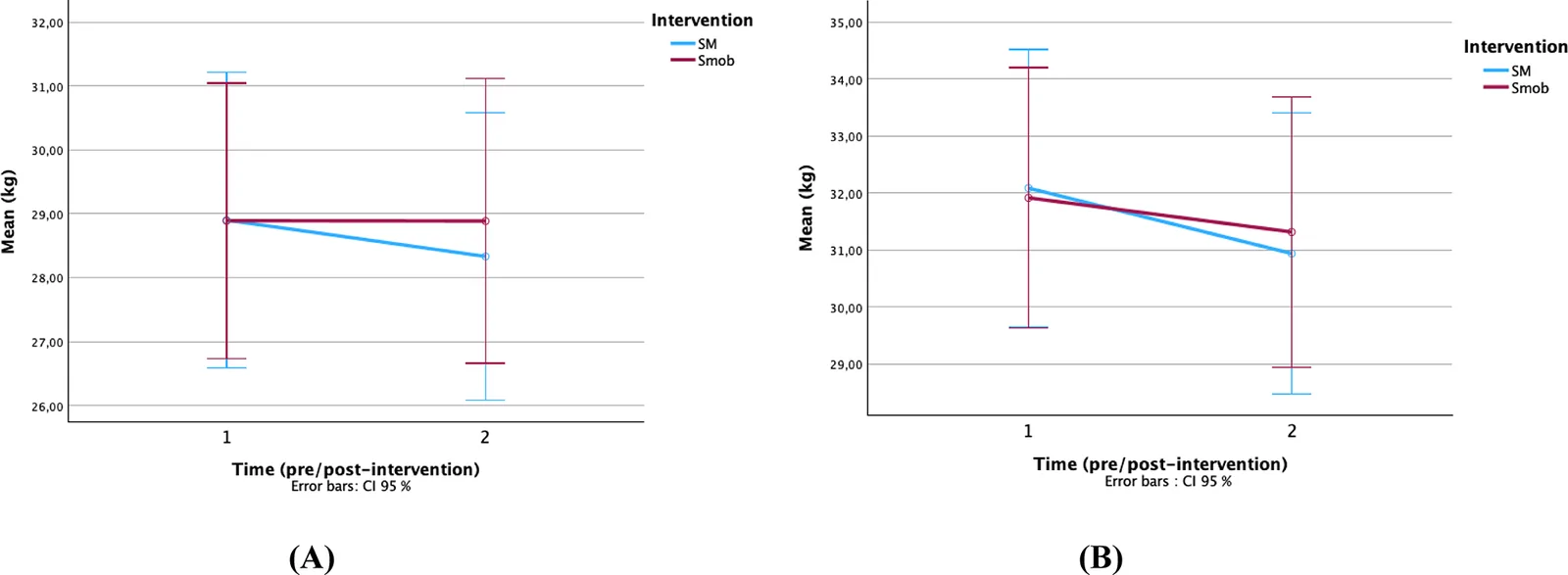
Left (**A**) and right (**B**) grip-strength pre- and post-intervention (SM vs Smob)*.* SM: Spinal Manipulation, Smob: Spinal mobilization, CI: confidence interval, kg: kilograms

On the right side, grip strength significantly decreased over time (main effect: *F* = 19.64, *p* < 0.001), with 1.13 kg after SM and 0.52 kg after Smob. These changes did not reach the minimal detectable change of 6 kg [46]. No significant difference between SM and Smob (main effect: *F* = 0.003, *p* = 0.955), nor any interaction effect was found (*F* = 3.07, *p* = 0.083). Sequence effect was not significant (*F* = 0.144, *p* = 0.706) but a significant period effect (*F* = 20.40, *p* < 0.001) was observed (See Fig. 7).

On the left side, grip strength did not significantly change over time (*F* = 1.74, *p* = 0.191), nor did it differ between SM and Smob (*F* = 1.16, *p* = 0.284), and no interaction effect was found (*F* = 3.26, *p* = 0.075). Sequence effect was not significant (*F* = 0.35, *p* = 0.555) but a significant period effect (*F* = 13.12, *p* < 0.001) was observed (See Fig. 7).

### Muscular response

Muscular response differed significantly between SM and Smob (Wilcoxon signed-rank test, p < 0.001). Specifically, muscular response (measured by nRMS) was higher after SM compared with Smob for both the SCM (0.144 ± 0.121 vs 0.023 ± 0.013) and UT (0.083 ± 0.100 vs 0.024 ± 0.025) muscles, on both sides. In contrast, a higher muscular response in favor of Smob was found for TA on both sides (0.017 ± 0.013 vs 0.029 ± 0.023) (see Table 5 for results). No significant sequence effect was observed for SCM (*U* = 282, *p* = 0.258), UT (*U* = 271, *p* = 0.184), or TA (*U* = 257, *p* = 0.114). A period effect was observed only for SCM with lower nRMS values during the second session compared with the first (*W* = 481, *p* = 0.038). No period effects were found for UT (*W* = 546, *p* = 0.133) or TA (*W* = 624, *p* = 0.418).

**Table 5:** nRMS values of muscular response (SCM, UT and TA) during SM and Smob

Muscle	Intervention	
	SM	SMob
SCM, mean (SD)	0.144 (0.121)	0.023 (0.013)
UT, mean (SD)	0.083 (0.100)	0.024 (0.025)
TA, mean (SD)	0.017 (0.013)	0.029 (0.023)

SM: spinal manipulation, Smob: spinal mobilization, SCM: sternocleidomastoid, UT: upper trapezius, TA: tibialis anterior, SD: Standard Deviation, nRMS: normalized root mean square

## Discussion

This mechanistic study sought to explore the differences between immediate clinical and neuromechanical effects of cervical SM and cervical Smob among patients with CNP. The results show differences in certain clinical and neuromechanical effects between SM and Smob. However, results showing statistical significance did not reach clinical significance and should therefore be interpreted with caution in the context of a mechanistic design.

### Pain intensity and ROM

Regarding pain intensity, results of this study show greater immediate decrease in pain intensity after SM compared to Smob, although MCID was not reached [33]. This is broadly in line with existing evidence [9–14]: a systematic review with meta-analysis by Wilhelm et al*.* [10], and a literature review by Giacalone et al*.*, reported improvements in pain intensity after a single or multiple SM sessions [13]. However, MCID was not consistently assessed or reached across the studies included in these reviews [10, 12, 13]. Moreover, Gorrell et al*.* reported a greater decrease in pain intensity after one SM at 7-day follow-up when compared to stretching as a control intervention [26]. In their meta-analysis, Gross and al. reported that SM was no more effective than Smob, either after one or multiple sessions, in improving pain intensity at short- and intermediate-term follow-ups [11].

The hypoalgesic effect of SM, discussed in several studies, is attributed to different peripheral and central mechanisms, but its clinical significance has yet to be determined [47–49]. It is possible that SM or Smob alone or a single session of each intervention may not be sufficient to achieve clinically relevant results for patients with CNP. For instance, in their randomized controlled trial, Farooq et al*.* found a clinically relevant improvement in pain intensity among individuals with chronic neck pain after 10 treatment sessions of mobilization combined with “routine physiotherapy”, compared to only “routine physiotherapy”, at four-week follow-up [50]. Additionally, Garcia-Gonzalez et al*.* reported a decrease in pain intensity exceeding MCID one week after one session of cervical or thoracic SM [51]. Therefore, clinical significance may have been reached in our study with more than one treatment session or with a multimodal approach as recommended in clinical practice [4–6, 13, 52]. Indeed, current clinical practice guidelines emphasize that multimodal approaches over several sessions and combining, for instance, exercises, manual therapy and patient education, are more effective than a single treatment approach in the context of CNP [4–6, 13, 52].

Our study shows an increase in all active ROM planes either after SM or Smob, which is consistent with current evidence [9–14]. Gross and al. reported in their meta-analysis that SM and Smob yielded similar improvement in ROM at short- and intermediate-term follow-ups [11]. While studies report increased ROM, various movement directions were investigated across studies. Specifically, Giacalone et al*.* reported an increase in extension and rotation [13] while Lascurain-Aguirrebeña et al*.* and Gorrell et al*.* reported an increase in lateral-flexion and rotation after one session of Smob [53] and one session of SM combined with stretching [26], respectively.

### Muscular response

This study is the first to compare individual effects of SM and Smob on neck muscles in the context of CNP. Our results show a higher muscular response during SM compared to Smob in SCM and UT on both sides, except for TA for which Smob led to a higher muscular response.

Previous studies have reported increases in EMG activity of several muscles in the thoracic and lumbar regions following either SM or Smob among symptomatic and asymptomatic individuals [12, 15–21]. For instance, Pagé et al*.* reported a muscular response of thoracic muscles two to three times higher during SM compared to Smob in healthy participants [17]. Lardon et al*.* confirmed these results as they found a higher muscular response of paraspinal muscles during SM compared to Smob among participants with chronic middle back pain [20]. Our results are also consistent with Gorrell et al*.* who found a higher EMG activity of cervical spine muscles (SCM and splenius cervicis) after a cervical SM among patients with mild neck disability [12].

The greater effect of SM on muscular response compared to Smob might partly be explained by the differences in force–time characteristics of the two interventions, particularly SM peak force and thus the rate of force application [17, 19, 20, 22]. Previous findings suggest a linear relationship between SM peak force and muscle activation both during and shortly after the thrust [19]. Specifically, higher SM peak forces have been associated with greater levels of muscle activation [17, 19, 20]. Given that peak forces and other force–time characteristics differ substantially between SM and Smob [35, 36], these biomechanical parameters likely account for a considerable portion of the observed differences in effects between the two interventions [17, 19–21].

In contrast, no clear physiological explanation currently accounts for the higher muscular response of TA observed during Smob. Moreover, the increase of electromyographic activity in TA was very low (∼3%) compared to that recorded in SCM (∼15%) and UT (∼8%) during SM.

### Pressure pain threshold

On one hand, our results show an increase in PPT for both right SCM and right UT after both interventions, with a greater increase after SM for the right SCM (SDD was not achieved) and on the other hand inconsistent decreases in PPT were recorded for the TA. In a systematic review, Millan et al*.* reported that most studies (19 of 27) observed a local increase in PPT at the site of intervention after cervical, thoracic and lumbar SM or Smob [23]. Moreover, Giacalone et al*.* and Lascurain-Aguirrebeña et al*.* reported an increase in PPT in the cervical region, either after cervical SM [13] or cervical Smob [24].

These changes could be explained by multiple neurophysiological mechanisms of SM and Smob [47–49]. In their narrative review, Gevers-Montoro et al*.* discussed several mechanisms by which SM and Smob might impact pain processing such as spinal segmental inhibition, decreasing peripheral pro-inflammatory responses and potentially supraspinal mechanisms that remain to be clarified [48].

Interestingly, Lardon et al*.* reported an immediate decrease in pressure-provoked pain intensity (which could be considered as an equivalent to an increase in PPT) after a thoracic SM, while Smob had no effect [20]. Additionally, in their study investigating PPT changes in “a real-world chiropractic setting”, Nim et al*.* reported no significant change in PPT after a chiropractic consultation [54]. This study included all types of chronic pain and different types of treatments, including SM, which makes it difficult to isolate SM or Smob effect on PPT.

Although some statistically significant effects were observed in our study, these patterns were not systematically replicated across bilateral muscle sites. For example, statistically significant effects identified in the right SCM were not consistently observed on the contralateral side, and while the right TA PPT values decreased over time following both interventions, the left TA showed an increase after SM and a decrease after Smob. Taken together, these inconsistent and hemilateral patterns may partly reflect statistical noise rather than intervention-specific physiological mechanisms. PPT findings should therefore be interpreted cautiously.

### Grip-strength

This study is, to our knowledge, the first to measure the effect of Smob on grip-strength and compare it to that of SM in the context of CNP. Our results are conflicting with previous studies regarding SM effects on grip-strength, as they show a decrease in grip-strength of the right hand after SM. However, these results should be interpreted with caution, as the decrease in grip-strength was minimal (1.13 kg on average) compared to the MDC threshold (6 kg). Moreover, some participants mentioned feeling discomfort while squeezing the hand-grip, which could have led to a lower score as the procedure was repeated.

In their pilot study with basketball players, Humphries et al*.* found no significant change in grip-strength after one single SM [55]. However, Botelho et al*.* reported an increase in grip-strength of both hands among judo athletes after a single and multiple SM sessions [56]. Similarly, Gorrell et al*.* observed an increase on the contralateral side in individuals with chronic neck pain following a single instrumented manipulation [26], and Bautista-Aguirre et al*.* reported increases in both hands after multiple SM sessions [27]. Nevertheless, these increases did not reach the MCID [26, 27] and in Botelho et al*.*’s study, clinical significance was not mentioned [56], questioning therefore their clinical relevance. In their study, Petersen et al. reported improvements in scapulothoracic muscle strength after a single session of cervical SM combined with an exercise program in individuals with neck pain compared with asymptomatic controls who received no intervention (lasting up to 96 h) [57]. However, the combination with an exercise program makes it difficult to attribute these changes specifically to SM.

The increase in grip-strength reported in some studies may be attributed to neurophysiological mechanisms. Several studies suggest that SM and Smob may engage corticospinal pathways, leading to modulation in motor unit recruitment and, consequently, in the voluntary activation of muscle fibers [58–61]. However, these neurophysiological responses remain insufficiently explored, especially in the context of specific musculoskeletal conditions. Furthermore, the lack of studies investigating the effect of Smob on grip-strength restricts direct comparisons with SM.

### Strengths and limitations

Few limitations need to be taken into consideration while interpreting the results of this study. First, although all participants reported chronic neck pain, symptom severity and disability levels were relatively mild in some individuals and may not fully represent a typical care-seeking clinical population with neck pain. For example, NDI scores at baseline show that 78.7% presented mild to moderate disability while 20.2% reported moderate to severe disability. Second, only immediate effects were assessed, which limits the ability to detect outcomes that may emerge over a longer follow-up period, as previously discussed regarding pain intensity. Third, despite randomization of treatment allocation, a 72-h washout period between sessions, and adjustment for period effects, residual carryover or temporal effects cannot be completely excluded and may have amplified or attenuated the observed treatment effects. Fourth, given the number of analyzed neuromechanical and clinical outcomes, the possibility of type I error cannot be excluded. As the present analyses were conducted within an exploratory mechanistic study, our findings should be interpreted cautiously. Fifth, considering the mechanistic nature of our study, SM and Smob were highly standardized across clinicians and participants. However, clinicians training and professional experience with each intervention varied across sites and may have influenced our findings. Finally, although ETS questionnaire results indicate similar expectations prior to each treatment, the inability to blind participants to the treatment received may have added a placebo effect [62, 63].

An important strength of this study is the direct comparison of clinical and neuromechanical effects between SM and Smob. Although MCID was only reached for flexion–extension and lateral-flexion ranges of motion, our results provide an understanding on how each manual therapy technique can yield distinct effects when studied separately. Another strength is the wide age-range of participants allowing for the generalizability of our results across different age groups (18 to 65 years old). Finally, as a multicenter study, therapists of diverse expertise and backgrounds were involved, which reflects the nature of musculoskeletal care, while acknowledging that mobilizations were not exclusively delivered by physiotherapists, the clinicians most commonly trained in this technique.

In the context of clinical practice, SM and Smob specific treatments effects are important to consider, as both treatments can be used and tailored to individual needs and clinical contexts when treating patients with CNP. The similarities between both manual therapy approaches suggest that manual therapy can be used and adapted to patients’ beliefs and preferences. While the mechanistic nature of our study precludes any conclusion regarding clinical supremacy of either treatment being made, their differences emphasize the importance of investigating the clinical and neuromechanical effects of each treatment. Future studies should address this topic while considering multiple sessions of each treatment.

## Conclusion

One treatment of cervical SM resulted in greater pain reduction and higher muscular response than cervical Smob in patients with CNP. Both interventions resulted in greater cervical ROM. However, results showing statistical significance did not reach clinical significance which suggests that the reader should refrain from drawing any conclusion regarding clinical practice.

The present findings contribute to the understanding of the immediate clinical and neuromechanical responses associated with cervical manual therapy interventions. However, given the mechanistic cross-over design, conclusions regarding clinical effectiveness and generalizability remain limited. Future clinical trials are required to determine the clinical relevance and longer-term implications of these findings.

## Data Availability

Data will not be made accessible due to ethics certification requirements.

## Abbreviations

CNP: Chronic neck pain
EMG: Electromyograhy.ic
ETS: Expectation of treatment scale
GBD: Global burden of disease
h: Hour.s
kg: Kilogram
MVC: Maximum voluntary contraction
NDI: Neck disability index
NPRS: Numerical pain rating scale
η^2^p: Partial eta squared
PGIC: Patient global impression of change
PPT: Pression pain threshold
ROM: Range of motion
SM: Spinal manipulation
Smob: Spinal mobilization
SCM: Sternocleidomastoid
TA: Tibialis anterior
UT: Upper trapezius
YLD: Years lived with disability
